# Glioblastoma pathophysiology: roles of aging driven changes in STAT3 interactions with NF-κB dimer components in the modulation of the mitochondrial melatonergic pathway and night-time inflammation resolution

**DOI:** 10.37349/etat.2026.1002358

**Published:** 2026-02-13

**Authors:** George Anderson

**Affiliations:** The First Clinical Medical College of Lanzhou University, China; CRC Scotland & London, Eccleston Square, SW1V 1PG London, UK

**Keywords:** glioblastoma, melatonin, STAT3, NF-κB, mitochondria, vagal nerve, *N*-acetylserotonin, treatment

## Abstract

Glioblastoma (GBM) is a complex condition with a poorly understood pathophysiology and no effective treatment to date. The present article highlights the role of canonical and non-canonical signal transducer and activator of transcription 3 (STAT3) interactions with nuclear factor kappa-light-chain-enhancer of activated B cells (NF-κB) in the modulation of the mitochondrial melatonergic pathway in GBM microenvironment pathophysiology. The capacity of STAT3 and NF-κB to interact to upregulate the mitochondrial melatonergic pathway is suppressed systemically over the course of aging, thereby attenuating the capacity to achieve inflammation resolution. The suppressed capacity to induce the mitochondrial melatonergic pathway systemically is partly driven by the dramatic 10-fold decrease in pineal melatonin over aging. The attenuation of pineal melatonin in the first half of sleep over aging and aging-accelerating conditions disinhibits the effects of cortisol in the second half of sleep. This decrease in the melatonin/cortisol ratio alters the nature of night-time dampening and resetting in preparation for the coming day by altering cellular and intercellular homeostatic interactions. Aging and aging-accelerating conditions, by impacting the night-time melatonin/cortisol ratio, also suppress the capacity of the vagal nerve to resolve inflammation. This further contributes to systemic changes that influence GBM pathoetiology and ongoing pathophysiology. Aging-associated changes in night-time dampening and resetting provide a novel framework on which many previously disparate bodies of data on GBM pathophysiology can be collated. This has numerous future research, prevention, and treatment implications.

## Introduction

There is a growing interest in how aging increases the risk of a host of diverse medical conditions, such as neurodegenerative disorders [1] and most cancers [2], including glioblastoma (GBM) [3]. GBM is a grade IV glioma, which is the most common and aggressive form of primary brain cancer in adults. GBM is genetically and phenotypically diverse, with rapid growth and spread. GBM is a poorly conceptualized and consequently poorly treated condition, with a risk that is increased over the course of aging and is significantly regulated by circadian processes [2, 3]. Aging is classically associated with increased oxidative stress that shortens telomeres and drives DNA damage, leading to DNA repair by the induction of poly-ADP-ribose polymerase 1 (PARP1). PARP1 deprives the cell of nicotinamide adenine dinucleotide (NAD^+^), thereby decreasing NAD^+^ dependent sirtuins. The suppression of sirtuins, especially sirtuin-3, increases oxidant production by the mitochondrial electron transport chain [4]. Telomere shortening and oxidative DNA damage and repair are classical cellular processes driven by oxidative stress that have long been proposed to underpin aging-associated changes, including in the course of GBM pathophysiology [5]. Telomere shortening, including by epigallocatechin gallate (EGCG), induces senescence and toxicity in GBM, highlighting the importance of oxidant regulation and NAD^+^ maintenance in GBM and GBM stem-like cell (GSC) survival [5]. Recent work has highlighted a wider circadian and systemic interaction in the modulation of aging-linked changes pertinent across diverse aging-linked medical conditions, including GBM [6].

Heightened levels of inflammation (inflammaging) and oxidative stress over aging may be importantly determined by alterations in night-time processes [7], which is powerfully driven by the 10-fold decrease in pineal melatonin between the second and ninth decade of life [8]. Melatonin kills most tumors as well as decreasing proliferation and metastasis, including in GBM [9]. As a powerful antioxidant and anti-inflammatory, the suppression of pineal melatonin at night over the course of aging contributes to disinhibiting GBM pathophysiological processes. However, pineal melatonin is only one aspect of circadian changes in inflammation regulation over the course of aging.

**Figure 1:**
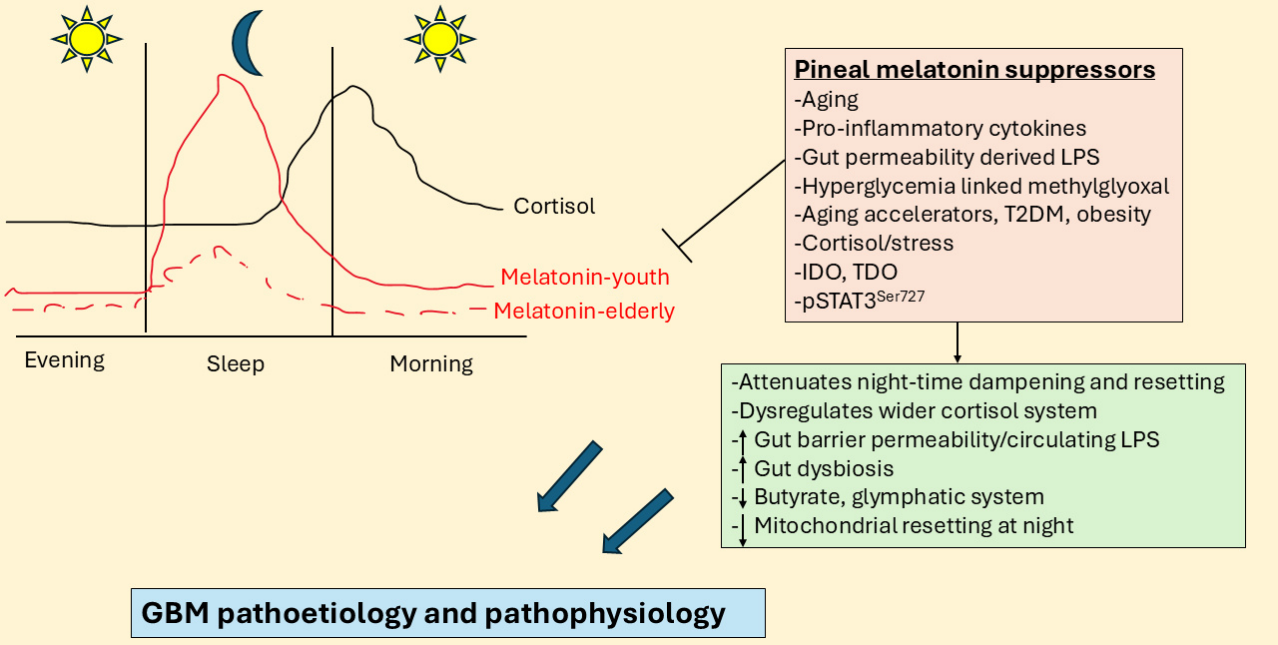
**Night-time cortisol and melatonin vary over age and by aging-linked processes.** The large blue arrows indicate that the interactions of pineal melatonin suppressors with their array of diverse consequences influence GBM pathoetiology and pathophysiology. GBM: glioblastoma; IDO: indoleamine 2,3-dioxygenase; LPS: lipopolysaccharide; pSTAT3: phosphorylated signal transducer and activator of transcription 3; T2DM: type 2 diabetes mellitus; TDO: tryptophan 2,3-dioxygenase.

Although relatively little is investigated and still poorly understood, there is a ‘stress-like’ rise in cortisol at night, peaking in the cortisol awakening response (CAR) in the first 30 min after awakening. Although typically seen as an awakening response, data on the morning CAR indicate that it is a circadian process [10]. The rise in cortisol shows a strong negative correlation with melatonin levels over the night (see Figure 1). This is likely to be of pathoetiological significance for a host of diverse aging-linked medical conditions, including GBM. Cortisol activation of the glucocorticoid receptor (GR)-α contributes to GBM pathophysiology, with raised cortisol and GR-α levels decreasing GBM patient survival [11]. Notably, melatonin suppresses cortisol production by the adrenal cortex [12], whilst also preventing activated GR-α nuclear translocation [13]. The aging-linked decrease in pineal melatonin interacts with night-time and morning CAR to alter how CNS and systemic cells, microenvironments, and systems are dampened and reset at night. This contributes to diverse aging-associated medical conditions, including GBM. Although typically modeled as a stress hormone, cortisol contributes to GBM pathophysiology [14], with circadian effects that may be disinhibited by the suppression of pineal melatonin [9, 10]. The gut microbiome and gut permeability are also integral aspects of the circadian rhythm [15]. Melatonin and cortisol have contrasting effects on the gut, indicating a role for alterations in night-time dampening and resetting in the modulation of gut dysbiosis/permeability [16, 17] over the course of aging and aging-associated medical conditions.

This article highlights the overlooked importance of aging-associated changes in night-time dampening and resetting in GBM pathogenesis and pathophysiology, highlighting the importance of the decreased night-time melatonin/cortisol ratio. It is proposed that this contributes to alterations in the local regulation of the mitochondrial melatonergic pathway by changing the levels and interactions of signal transducer and activator of transcription 3 (STAT3) with nuclear factor kappa-light-chain-enhancer of activated B cells (NF-κB), thereby limiting the availability of local melatonin in the course of inflammation resolution. Inflammation resolution is a core aspect of most medical conditions, including GBM. The article therefore provides a novel framework on which to integrate wider bodies of data on GBM pathophysiology, with novel research, treatment, and preventative implications.

## Gut microbiome interactions with the circadian rhythm

As with most medical conditions, alterations in the gut microbiome, including decreased short-chain fatty acids (butyrate, acetate, and propionate), are relevant aspects of GBM pathophysiology. Preclinical models provide strong evidence for the utility of sodium butyrate in GBM-glioma models, where butyrate promotes glioma cell apoptosis, decreases proliferation, and disrupts the cell cycle [18]. As butyrate can upregulate the melatonergic pathway, as shown in other cell types [19], butyrate effects may be partly mediated by increased melatonergic pathway induction in GBM and/or GBM microenvironment cells. Butyrate induced melatonin increases microRNA (miR)-138 to suppress programmed cell death (PD)-1 expression [7], with miR-138 significantly down regulated in GBM [20]. The regulation of miR-138 suppression of PD-1 by pineal and local melatonin in GBM microenvironment cells requires investigation.

As with melatonin, butyrate suppresses GR-α nuclear translocation [19], indicating GR-α nuclear translocation inhibition by butyrate at night and during the morning CAR, as well as in the course of daytime stress induced cortisol. By inhibiting GR-α nuclear translocation, both butyrate and melatonin alter the consequences of circadian and stress-linked hypothalamic-pituitary-adrenal (HPA) axis activation. Butyrate and melatonin, as well as directly regulating the GBM microenvironment, can also alter the consequences of stress and circadian cortisol and therefore the role of cortisol and the GR in GBM pathophysiology [11].

The suppression of pineal melatonin over aging contributes to increasing gut permeability/dysbiosis, thereby decreasing butyrate levels. Suppressed melatonin and associated alterations in gut microbiome products over aging therefore contribute to aging-linked medical conditions, including GBM. Pineal melatonin suppression over aging alters the wider cortisol ‘system’ including GR levels, subtypes (GR-α, GR-β) and GR sites of expression (plasma membrane, mitochondrial membrane, mitochondrial matrix, and cytoplasm). The suppression of melatonin at night over aging may therefore be intimately associated with significant changes in the wider cortisol ‘system’ and therefore with driving GBM pathophysiology.

## The mitochondrial melatonergic pathway in the GBM microenvironment

**Figure 2:**
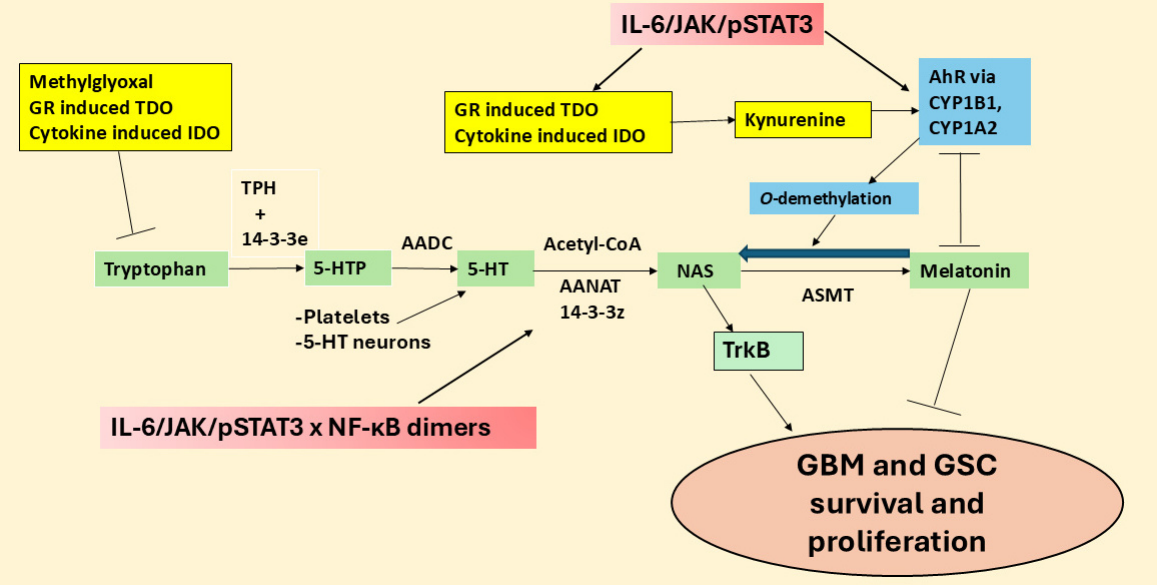
**The tryptophan-melatonin pathway and its regulators in GBM.** 5-HT: serotonin; 5-HTP: 5-hydroxytryptophan; AADC: aromatic-L-amino acid decarboxylase; AANAT: aralkylamine *N*-acetyltransferase; AhR: aryl hydrocarbon receptor; ASMT: *N*-acetylserotonin *O*-methyltransferase; CYP: cytochrome P450; GBM: glioblastoma; GR: glucocorticoid receptor; GSC: glioblastoma stem-like cell; IDO: indoleamine 2,3-dioxygenase; IL: interleukin; JAK: Janus kinase; NAS: *N*-acetylserotonin; NF-κB: nuclear factor kappa-light-chain-enhancer of activated B cells; pSTAT3: phosphorylated signal transducer and activator of transcription 3; TDO: tryptophan 2,3-dioxygenase; TPH: tryptophan hydroxylase; TrkB: tyrosine receptor kinase B.

There is an increasing interest in the regulation and role of the mitochondrial melatonergic pathway (see green shade, Figure 2), which seems to be an under-investigated core process in possibly all mitochondria-containing cells, with relevance across diverse medical conditions [1]. The melatonergic pathway is evident in all human cells so far investigated, including neurons and astrocytes [21], and potentially in the cells of all forms of multicellular life on planet Earth [22]. This may be especially important in cancer and other proliferative conditions, given that the two products of the melatonergic pathway, melatonin and its precursor, *N*-acetylserotonin (NAS), have opposing effects on GBM survival and proliferation [23]. This arises partly from NAS being a brain-derived neurotrophic factor (BDNF) mimic via activation of the BDNF receptor, tyrosine receptor kinase B (TrkB) [24]. TrkB activation increases the survival and proliferation of GSC [25]. The regulation of the melatonergic pathway, especially the NAS/melatonin ratio, may therefore be of considerable importance in GBM pathoetiology and pathophysiology, including from NAS and melatonin release by other cells of the GBM microenvironment [26]. The aryl hydrocarbon receptor (AhR) is a major determinant of the NAS/melatonin ratio via AhR-induction of cytochrome P450 (CYP)1B1 and CYP1A2, which can ‘backward convert’ melatonin to NAS via *O*-demethylation, as well as significantly decreasing melatonin effects by increasing its hydroxylation [23, 26]. The AhR is typically seen as a significant target for activation by most tumors via AhR activation suppressing natural killer (NK) cells and CD8^+^ T cells [23, 26]. The targeting of the AhR by tumor cells via kynurenine release may also be acting on the melatonergic pathway, including by increasing the NAS/melatonin ratio. The melatonergic pathway and its regulators in GBM are shown in Figure 2.

## STAT3 and NF-κB dimer components interact to regulate the melatonergic pathway

**Figure 3:**
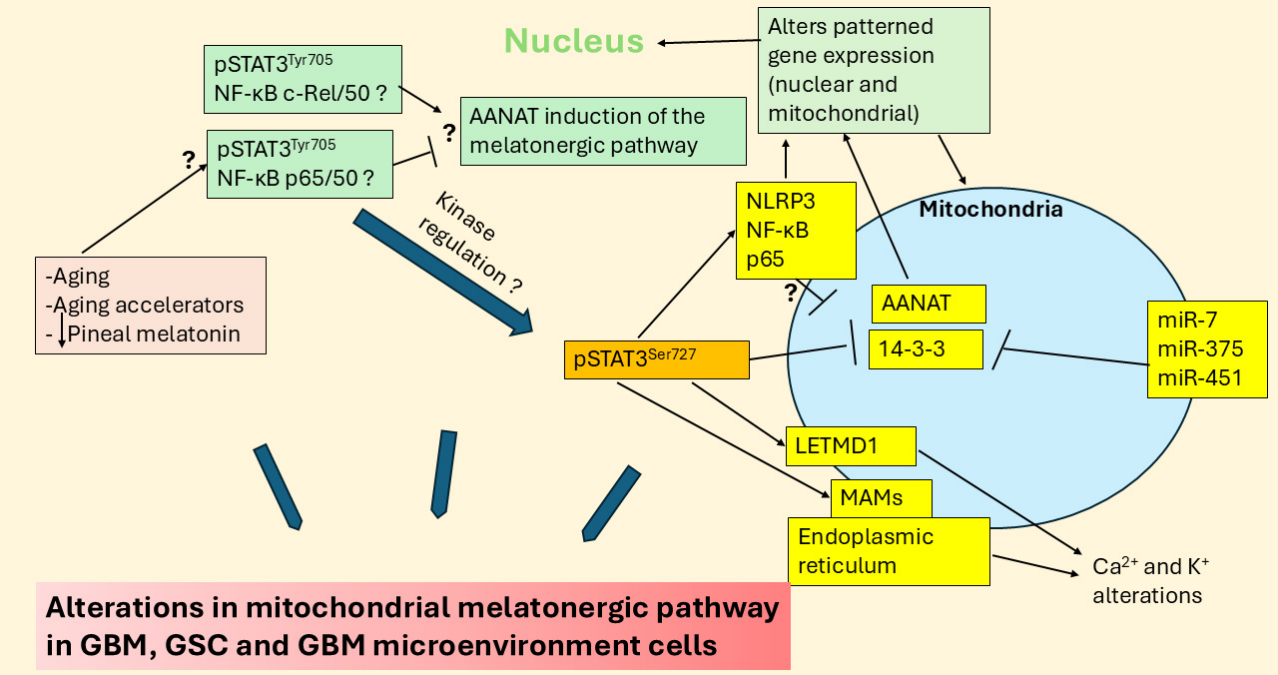
**Aging, aging accelerators, and decreased pineal melatonin may modulate STAT3 interactions with NF-κB dimer composition in regulation of the mitochondrial melatonergic pathway and wider GBM regulators.** The large blue arrows indicate that the variable influence of different factors and their interactions in the regulation of the melatonergic pathway in GBM, GSC, and GBM microenvironment cells. AANAT: aralkylamine *N*-acetyltransferase; GBM: glioblastoma; GSC: glioblastoma stem-like cell; LETMD1: leucine zipper EF-hand containing transmembrane protein-domain containing 1; MAMs: mitochondria-associated membranes; miR: microRNA; NF-κB: nuclear factor kappa-light-chain-enhancer of activated B cells; NLRP3: NLR family pyrin domain containing 3; pSTAT3: phosphorylated signal transducer and activator of transcription 3; STAT3: signal transducer and activator of transcription 3.

Recent work indicates that the regulation of local melatonin production in cells is intimately linked to how STAT3 interacts differentially with the various components of the NF-κB dimer. STAT3 and NF-κB dimer composition interact to either induce or suppress the mitochondrial melatonergic pathway [27]. The phosphorylated STAT3 (pSTAT3) leads to its activation, with tyrosine705 phosphorylation leading to pSTAT3^Tyr705^ nuclear (canonical) translocation and pSTAT3^Ser727^ phosphorylation leading to mitochondrial (non-canonical) translocation. Mitochondria translocating pSTAT3^Ser727^ is proposed to lead to pSTAT3 interacting with 14-3-3 to suppress the availability of mitochondrial 14-3-3 to stabilize the first enzyme of the melatonergic pathway, aralkylamine *N*-acetyltransferase (AANAT), which in the presence of acetyl-coenzyme A converts serotonin (5-HT) to NAS [23], as shown in Figure 2. Both nuclear and mitochondrial pSTAT3 (canonical and non-canonical, respectively) may therefore regulate the mitochondrial melatonergic pathway. This is of importance as the raised pSTAT3 levels in GBM are widely recognized as significant aspects of a number of GBM pathophysiological processes and a significant treatment target. For example, pSTAT3^Ser727^ forms a positive feedback loop with leucine zipper EF-hand containing transmembrane protein-domain containing 1 (LETMD1) to regulate mitochondrial Ca^2+^ and K^+^, further altering core aspects of mitochondrial function [28, 29]. GBM also effluxes interleukin (IL)-11 to increase pSTAT3 in astrocytes that induces T cell apoptosis via astrocyte release of the death receptor ligand, tumor necrosis factor-related apoptosis-inducing ligand [30]. This indicates that the induction of pSTAT3 in astrocytes is a significant aspect of how GBM controls the immune response, but also indicates that GBM fluxes can control the astrocyte melatonergic pathway. Putative canonical and non-canonical STAT3 interactions with NF-κB dimer composition in GBM and GBM microenvironment cells are shown in Figure 3.

Aging-linked alterations in how cells, cell microenvironments, and body systems are dampened and reset at night may gradually change over the course of aging and by aging accelerators, such as type 2 diabetes mellitus (T2DM), obesity, and stress [1]. This is at least partly mediated by decreased pineal melatonin that contributes to GBM pathoetiology and pathophysiology, as in many other aging-associated conditions [1]. The loss of pineal melatonin is a significant change over the course of aging. However, the role of pSTAT3 (nuclear and mitochondrial) interactions with NF-κB dimer composition in regulating the local melatonergic pathway within GBM microenvironment cells is an under-researched and possibly core aspect of GBM pathophysiology [7]. The relevance of local melatonergic pathway regulation is supported by data showing exogenous melatonin suppresses pSTAT3 in GSC, leading to decreased initiation and propagation [31]. This indicates a circadian processes (pineal melatonin) may modulate the local melatonergic pathway (via pSTAT3-NF-κB dimer composition), thereby allowing circadian/systemic processes to modulate and reset core cellular processes across systemic cells, microenvironments, and systems. This provides a novel perspective that links diverse and previously disparate data on GBM pathophysiology, including the role of increased aerobic glycolysis and hyperglycemia driven methylglyoxal in GBM. Methylglyoxal binds tryptophan via protein-protein interactions to suppress tryptophan availability for tryptophan-melatonin pathway initiation across cells in the GBM microenvironment, including astrocytes [32]. Methylglyoxal also acts as a precursor for advanced glycation end products (AGEs) that activate the receptor for AGEs (RAGE), which upregulates pSTAT3 [33]. Methylglyoxal therefore may suppress the availability of cellular melatonin by a number of routes, by binding tryptophan and RAGE upregulation of pSTAT3. This would indicate that GBM upregulation of glycolysis is not simply an additional energy source but is also a significant modulator of core cellular processes pertinent to GBM initiation and proliferation, including the mitochondrial melatonergic pathway. The circadian changes underpinning this are overviewed next.

## Night-time dampening and resetting

Melatonin levels and cortisol effects may vary over the course of aging, as highlighted by typical values in Figure 1 [7, 34]. Figure 1 clearly shows the dramatic decrease in pineal melatonin evident between the second (youth) and ninth (elderly) decade of life. This significant decrease in pineal melatonin at night in the elderly is not replicated by alterations in the night-time rise in cortisol. As melatonin suppresses GR-α, the loss of melatonin initially disinhibits cortisol at the GR-α, which may then parallel a stress response that leads to wider dysregulation of the cortisol system, including GR-β upregulation as well as changes in GR localization to the plasma membrane, mitochondrial membrane and/or mitochondrial matrix, thereby allowing cortisol to have diverse effects dependent upon GR receptor subtype and localization site. The lost priming and dampening effect of relatively high pineal melatonin levels is lost over aging, with many consequences, including in cortisol effects.

The suppressed capacity of pineal melatonin to dampen any residual inflammation at a given site may also act to ‘flag’ that site for cortisol to dampen inflammation in the second half of sleep and during the morning CAR. As noted, raised cortisol and GR-α levels enhance GBM proliferation and decrease patient survival [11]. Whether melatonin and butyrate suppress not only the GR-α nuclear translocation but also modulate GR mitochondrial translocation to the mitochondrial membrane and/or the mitochondrial matrix and/or plasma membrane still requires investigation. GR mitochondrial translocation, either to the mitochondrial membrane and/or the mitochondrial matrix will be important to determine in future research, given their dramatic effects on mitochondrial function. Local cortisol production by 11β-HSD1 is predominantly driven by local pro-inflammatory cytokines, which are disinhibited by attenuated melatonin levels in the course of inflammaging [35]. CNS 11β-HSD1L is a significant pathophysiological factor in GBM [36, 37].

As shown in Figure 1, there is a dramatic decrease in pineal melatonin production over aging. This also has consequences for night-time cortisol and morning CAR rise, given that melatonin, like gut microbiome-derived butyrate, suppresses GR-α nuclear translocation. Pineal melatonin in the first half of sleep also has priming effects on cells, microenvironments, and body systems that optimize mitochondrial function and change the consequences of cortisol production over the night and during the morning CAR, with this priming effect significantly altered over the course of aging. The loss of melatonin either from aging or from aging accelerating conditions, such as T2DM, obesity, hypertension, and night-shift work, contributes to cancer and other aging-associated medical conditions, including neurodegenerative conditions [1] and cardiovascular disease [38]. As noted, the suppression of pineal melatonin over aging and aging-accelerating conditions will have consequences for the gut, including increasing gut dysbiosis and permeability. This is coupled to a decrease in butyrate and an increase in circulating lipopolysaccharide (LPS), which modulates both GBM and GBM microenvironment cells [39]. Both melatonin and butyrate, as a histone deacetylase inhibitor (HDACi), induce GBM apoptosis and decrease proliferation [17], whilst enhancing the cytotoxic efficacy of NK cells [40, 41] and modulating other immune cells [42, 43], the loss of butyrate and melatonin over aging changes the homeostatic interactions of tumor microenvironment cells. The consequences of decreased pineal melatonin and gut microbiome butyrate in the modulation of cortisol effects in GBM are highlighted next.

## Cortisol and GBM

The circadian regulation of cortisol, and the putative disinhibited influence of cortisol and its receptors at different cellular localization sites over the course of aging, seems intimately associated with GBM growth [11]. These authors showed that daily glucocorticoids can enhance or inhibit GBM growth via GR signaling, with these opposing effects dependent upon the time of day of administration as well as the clock genes, *Bmal1* and *Cry*. This investigation concludes that murine and human GBM have an intrinsic clock gene circadian rhythm in vitro and in vivo that entrains to the host via cortisol/GR signaling, with this occurring regardless of GBM subtype or host immune status [11]. GBM therefore entrains to the brain circadian circuit, with growth modulated by clock-controlled cues, such as cortisol/GR and the loss of pineal melatonin over aging. Such data highlights the importance of the circadian rhythm to GBM initiation and ongoing homeostatic interactions in the GBM microenvironment and therefore the influence of aging-associated changes in night-time circadian rhythm regulation.

Although mitochondrial function has been investigated in GBM, with enhanced mitochondrial transcription elongation factor (TEFM), inducing malignant progression [44], the impact of cortisol on mitochondrial function or GR expression at the mitochondrial membrane still requires investigation. The wider cortisol system (namely plasma membrane GR-α, mitochondrial membrane or matrix GR-α and/or GR-β, and 11β-HSD1) have not been extensively investigated in GBM and the GBM microenvironment. It is highly likely that the suppression of pineal melatonin and gut butyrate over aging will disinhibit this wider cortisol system, with a potentially diverse array of cortisol effects at different GR subtypes and localization sites across the diverse cells in the tumor microenvironment.

In rodents, GBM show increased proliferation following castration, which is proposed to be mediated by heightened HPA axis induction and GR activation [45]. These authors also show that heightened GR activation enhances the growth and proliferation of non-GBM cancers inserted into the CNS [45]. This suggests that CNS processes in the wider tumor microenvironment may be importantly modulated by heightened GR activation [45]. Whether EGCG, which inhibits 11β-HSD1 by protein-protein interactions [46], modulates the influence of decreased testosterone over aging via GR potentiation of GBM initiation and proliferation will be important to determine. As 11β-HSD1 also regulates the induction of 11-oxygenated androgens such as 11-ketotestosterone (11KT), by aldo-keto reductase 1C3, the inhibition of 11β-HSD1 increases 11KT and therefore circulating androgens, as shown in T2DM patients [47]. 11KT is a potent androgen receptor agonist and the major circulating androgen in castration-resistant prostate cancer patients. EGCG suppression of 11β-HSD1 may therefore also upregulate 11KT, thereby attenuating the systemic impact of suppressed testosterone induced HPA axis activation over the course of aging. This may be parsimonious with EGCG affording protection against GBM induction [48], whilst also increasing apoptosis [5, 49].

GR activation also increases leucine-rich repeat kinase 2 (LRRK2) and α-synuclein, as shown in neurons [50], with LRRK2 contributing to stem-like qualities in GBM [51], whilst α-synuclein is released by GBM and ‘spreads’ to astrocytes, where it contributes to the induction of stem-like qualities in astrocytes [52]. GR induction of LRRK2 and α-synuclein may therefore be involved in stem cell-like transformation in the GBM microenvironment as well as within GBM. Notably, melatonin can suppress both LRRK2 and α-synuclein induction and aggregation [53, 54]. α-Synuclein can also suppress NAS *O*-methyltransferase (ASMT) and therefore melatonin production by increasing the binding of microtubule-associated protein 1 light chain 3 beta (LC3B) to ASMT, thereby decreasing the conversion of NAS to melatonin [55]. Melatonin and α-synuclein therefore have negative reciprocal interactions as well as contrasting effects in GBM. Should α-synuclein suppress ASMT in astrocytes that neighbor GBM, astrocytes would then be more likely to efflux NAS [56], rather than melatonin [21, 57], thereby activating TrkB to further contribute to the induction, survival, and proliferation of GSC [23, 58]. The capacity of GR activation to induce α-synuclein may therefore contribute to accelerated metabolism of ASMT to increase the NAS/melatonin ratio and GSC proliferation.

**Figure 4:**
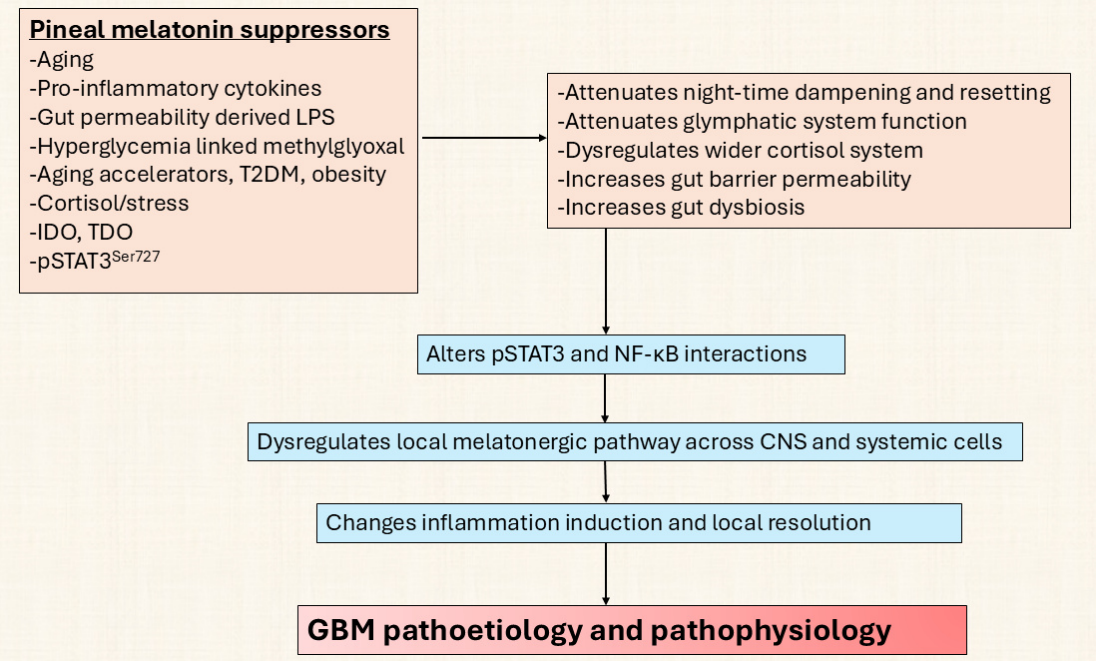
**Cortisol and α-synuclein suppress ASMT to increase NAS and GBM, GSC proliferation.** The large blue arrows indicate that the variable influence of different factors and their interactions in the regulation of GBM and GSC proliferation and survival. ASMT: *N*-acetylserotonin *O*-methyltransferase; GBM: glioblastoma; GR: glucocorticoid receptor; GSC: glioblastoma stem-like cell; NAS: *N*-acetylserotonin; TEFM: mitochondrial transcription elongation factor.

The effects of α-synuclein in GBM and GBM microenvironment cells may parallel those of TEFM, which also increases GBM proliferation and survival [44] as well as promoting autophagy/LC3B. This could suggest that TEFM upregulation of autophagy/LC3B, may upregulate the NAS/melatonin ratio with benefits for GBM and GSC survival, proliferation, and metastasis via released NAS induced TrkB activation, as shown in other tumor cells [59]. The GR translocation to the mitochondrial membrane regulates mitochondrial gene transcription directly and indirectly, including TEFM [60], suggesting that dysregulation of GR sites of expression may be linked to TEFM/LC3B, leading to suppressed ASMT and increased NAS/melatonin ratio. The changes in the wider cortisol system may therefore be influenced by suppressed pineal melatonin efflux, with consequences arising from altered GR signaling that decreases local melatonin availability (see Figure 4).

## Night-time melatonin and cortisol in glymphatic system regulation

The suppression of pineal melatonin and the heightened influence of cortisol over the course of aging is relevant to the pathoetiology and ongoing pathophysiology of a host of diverse medical conditions, including Alzheimer’s disease [1], cardiovascular diseases [61], and cancer [7]. There is a growing appreciation that an important aspect of aging-associated CNS disorders arises from the attenuated function of the glymphatic system [62]. The glymphatic system is the debris clearing process in the CNS that is powerfully determined by the polarization of astrocytes via aquaporin 4 (AQP4) being expressed on astrocytic end-feet [62]. Tanycytes can provide a similar role, especially in the hypothalamus [63]. Importantly, the glymphatic system is primarily circadian regulated with most brain debris cleared at night, which is potentiated by pineal (and possibly astrocyte) melatonin [64], whilst enhanced stress driven cortisol effects at the GR-α generally suppress the glymphatic system [65]. Clearly, the dramatic decrease in the night-time melatonin/cortisol ratio over the course of aging has detrimental effects on glymphatic system function, leading to the accumulation of debris that increases the risk of aging-associated CNS medical conditions, including GBM [66].

Exploration of the glymphatic system and closely associated meningeal lymphatic system in GBM is still in its earliest stages. A recent systematic review highlighted attenuated lymphatic outflow and disrupted fluid drainage in GBM, with novel implications for GBM management [67]. These authors also highlighted the negative implications that a suppressed glymphatic system function has for drug delivery and immunotherapy, as well as imaging interpretation [67]. Data also shows tumor volume to inversely correlate with glymphatic function, with astrocyte AQP4 levels positively correlated with peritumoral brain edema volume, whilst inversely correlating with proton density in peritumoral brain edema areas [68]. The suppression of the glymphatic system is also evident in GBM preclinical models [69], which the authors suggest may lead to the accumulation of toxic waste and pro-inflammatory factors that may contribute to GBM pathoetiology. An accumulation of alterations in night-time processes therefore seems intimately linked to GBM susceptibility and pathophysiology, which is further indicated by shorter sleep time increasing GBM risk [3].

T2DM is another condition associated with decreased pineal melatonin as well as increased gut permeability and gut dysbiosis [70]. Most data show T2DM to be a GBM risk factor [71]. Many of the detrimental effects of T2DM are mediated by hyperglycemia driven methylglyoxal. Methylglyoxal suppresses the melatonergic pathway via at least two routes, namely: 1) protein-protein interactions with tryptophan [32] and 2) by methylglyoxal being a precursor for AGEs that activate the RAGE, which changes the interactions of STAT3 and NF-κB dimer components [72]. This is covered in more detail below, but it is important to highlight as this is a relevant aspect of alterations in night-time processes and accelerated aging, which also negatively regulate glymphatic system function. Methylglyoxal effects also have treatment and preventive implications as quercetin quenches methylglyoxal [73] and therefore prevents the methylglyoxal suppression of the tryptophan-melatonin pathway. Quercetin has been extensively proven to have utility in GBM management [74], and whether this is at least partly mediated by upregulation of the tryptophan-melatonin pathway and local melatonin production in GBM microenvironment cells will be important to determine in future research. One preclinical study indicates that the glymphatic system shows no circadian regulation [75], suggesting that the capacity of astrocytes [21] and other GBM microenvironment cells to upregulate the tryptophan-melatonin pathway, and therefore melatonin’s optimization of glymphatic system function, may determine glymphatic system function irrespective of the circadian rhythm. If so, this would indicate the importance of achieving local resolution of inflammatory activity via local melatonin efflux in the regulation of the glymphatic system.

**Figure 5:**
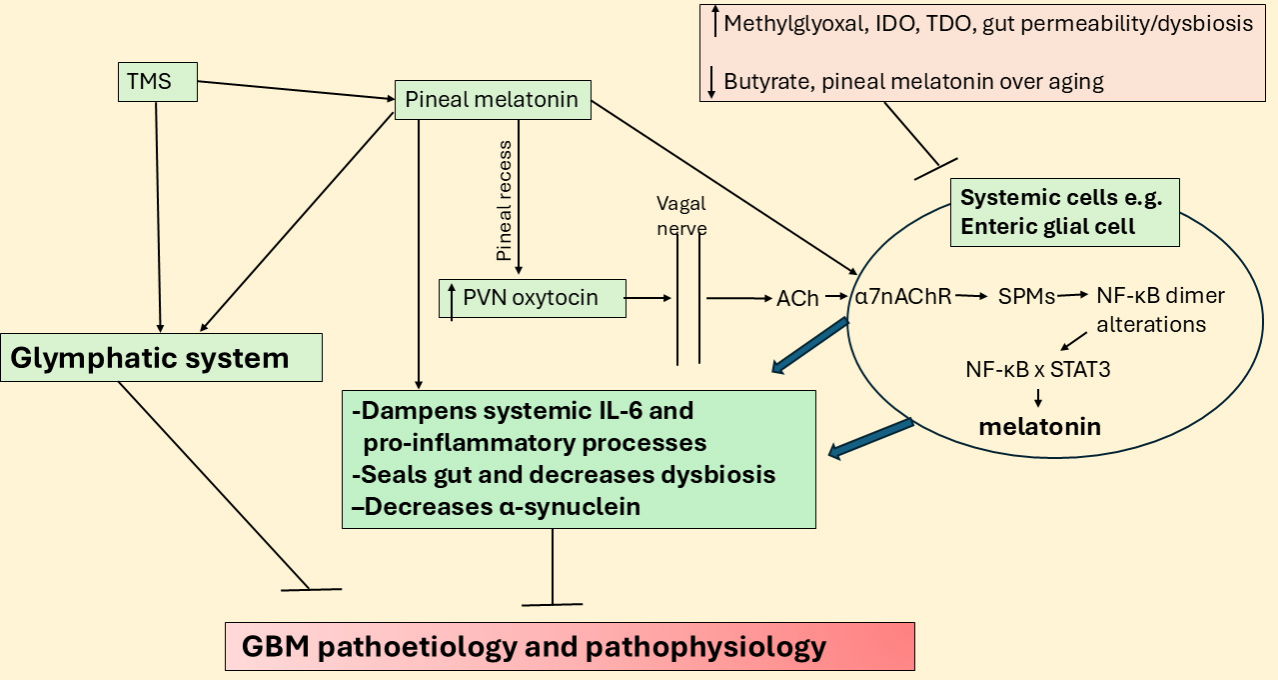
**Suppressed pineal melatonin modulates gut products to suppress the glymphatic system and increase GBM pathoetiology and pathophysiology.** AQP4: aquaporin 4; GBM: glioblastoma; IDO: indoleamine 2,3-dioxygenase; LPS: lipopolysaccharide; pSTAT3: phosphorylated signal transducer and activator of transcription 3; T2DM: type 2 diabetes mellitus; TDO: tryptophan 2,3-dioxygenase; TMAO: trimethylamine-*N*-oxide.

Closely interconnected with glymphatic system function is the recently recognized role of the meningeal lymphatic system in the modulation of GBM and wider brain processes [76]. Interestingly, gut microbiome derived products, including butyrate [77] and trimethylamine-*N*-oxide modulate the glymphatic system and meningeal lymphatic system [78]. Such data highlights the role of wider systemic processes and their alterations by aging-associated changes in the modulation of the CNS circadian rhythm. This is a two-way interaction given the differential effects on the circadian melatonin/cortisol ratio on the gut microbiome/permeability at night (see Figure 5).

## Cyclooxygenase-2, NF-κB, and STAT3

A growing body of data highlights the role of cyclooxygenase (COX)-2 in the pathophysiology of GBM [79, 80]. NF-κB can induce COX-2 in GBM [81], whilst COX-2 induced prostaglandin (PG)E2 feeds back on NF-κB to either suppress and/or alter the composition of the NF-κB dimer components, including upregulating the pro-inflammatory p65, as shown in other cancer cells [82]. COX-2 also contributes to the immunosuppressive microenvironment, with effects primarily investigated in macrophages [83]. This is relevant to temozolomide treatment resistance, which is at least partly mediated by increased COX-2 in GBM being released via extracellular vesicles to change the tumor microenvironment [83]. The COX-2 inhibitor, celecoxib, increases temozolomide efficacy in GBM cell lines [84], with implications for how GBM, including via extracellular vesicles containing COX-2, regulates the wider tumor microenvironment. Celecoxib also suppresses PD-ligand 1 (PD-L1) levels in GBM, highlighting its impact on the tumor microenvironment interactions, with effects that are mediated via increased FKBP prolyl isomerase 5 [85], and therefore associated with an attenuation of cortisol effects at the GR [86]. COX-2 and celecoxib effects are complicated by their regulation of STAT3 and therefore the interface of STAT3 with NF-κB dimer components, AANAT, and the melatonergic pathway.

COX-2 can modulate STAT3 via a number of mechanisms, including the induction of IL-6 and therefore the IL-6/Janus kinase (JAK)/pSTAT3 pathway [87] as well as the COX-2/PGE2/EP4 induction of STAT3 as shown in hepatocellular carcinoma cells [88] and colorectal cancer cells [89]. NF-κB and pSTAT3 may also interact to induce the COX-2/PGE2/EP4 pathway, as shown in pancreatic cancer [90]. The efficacy of celecoxib across a number of medical conditions involves the suppression of STAT3 [91], including in esophageal squamous cell carcinoma [92] and the cancer stem-like cells of medulloblastoma [93]. Given the importance of pSTAT3 interactions with NF-κB dimer composition in the induction or suppression of the melatonergic pathway, the regulatory interactions of COX-2 with STAT3 and NF-κB would indicate a role for COX-2 in the modulation of the melatonergic pathway. Melatonin inhibits COX-2, including when induced by a leaky gut linked increase in circulating LPS [94], with melatonin effects mediated by the inhibition of NF-κB p50/p50 induction of COX-2 [95]. Such suppressive effects of melatonin on COX-2 occur without some of the side-effects associated with pharmaceutical COX-2 inhibitors such as celecoxib [96]. Overall, COX-2 is closely associated with the regulation of the melatonergic pathway, and this association requires investigation in GBM and GBM microenvironment cells.

Another receptor associated with GBM proliferation and melatonergic pathway suppression is the purinergic P2X7 receptor (P2X7r).

## Purinergic P2X7 receptor

Purinergic P2X7r activation enhances GBM growth and proliferation following raised microglia and macrophage ATP efflux. P2X7r antagonism decreases GBM proliferation and cell survival [97], including by the depletion of GSC [98]. The P2X7r regulates a wide array of diverse cancers, including pancreatic and lung cancer, as well as hepatocellular carcinoma, leukemia, and lymphoma [99]. P2X7r antagonism is also a significant treatment target in Alzheimer’s disease [100], retinal diseases [101], PTSD [102], cardiovascular diseases [103], autism [104], long COVID [105], depression [106], and pulmonary hypertension [107]. P2X7r effects are proposed to be mediated by a diverse array of disease-specific mechanisms, as well as via the modulation of the gut microbiome [108], and the P2X7r induction of the NLR family pyrin domain containing 3 (NLRP3) inflammasome [109]. The wide array of current medical classifications that the P2X7r modulates is partly reflective of its diverse expression across different cell types, including immune cells.

Such diverse P2X7r effects may also arise from mitochondria modulation, where P2X7r activation can increase reactive oxygen species (ROS) [110] to trigger NLRP3 inflammasome activation among many other processes. The P2X7r, like TrkB, GR, and melatonin receptors, is also expressed on the mitochondrial membrane, indicating a more direct association with mitochondrial function [111]. Pertinent to this, P2X7r activation suppresses pineal melatonin production from NAS, thereby increasing the pineal NAS/melatonin ratio [112, 113]. As noted above, NAS, via TrkB activation, can increase GSC survival and proliferation [58]. Whether P2X7r activation upregulates the NAS/melatonin ratio in GBM and other cells of the GBM microenvironment requires investigation.

P2X7r activation may modulate the melatonergic pathway via a number of mechanisms, including via STAT3 induction and IL-6/JAK/pSTAT3 pathway upregulation, as evident in many cancer types [114]. P2X7r upregulation of IL-6 also increases indoleamine 2,3-dioxygenase (IDO) and the conversion of tryptophan to kynurenine, which activates the AhR to increase the NAS/melatonin ratio [115]. Astrocyte, microglia and macrophage P2X7r activation increases IL-6 release [116], indicating alterations in the intercellular interaction of the GBM microenvironment, including the release of ATP and P2X7r activation. IL-6 is significantly increased in the GBM microenvironment, where it negatively correlates with patient survival time and may determine the processes underpinning GBM microenvironment changes, including immunosuppression [117]. The effects of P2X7r activation may therefore be importantly determined by the Ca^2+^/ROS driven IL-6 increase that upregulates the JAK/pSTAT3 pathway, thereby plausibly paralleling COX-2 effects via pSTAT3 interactions with NF-κB dimer components in the modulation of the mitochondrial melatonergic pathway.

This is supported by P2X7r induction of NF-κB, including in astrocytes where it enhances NF-κB p65 nuclear translocation [118]. P2X7r activation also enhances toll-like receptor 4 (TLR4) induction of NF-κB [119], allowing a leaky gut derived circulating LPS [120] and/or endogenous TLR4 ligands, such as high mobility group box 1 (HMGB1) [121], via TLR4 activation, to modulate the P2X7r effects in GBM microenvironment cells [122]. Notably, the TLR4/NF-κB p65 pathway increases beta amyloid cleaving enzyme 1 to increase amyloid-β and associated hyperphosphorylated tau in GBM [123, 124]. Although amyloid-β and hyperphosphorylated tau are classically linked to Alzheimer’s disease pathophysiology, their increase in GBM is predictable as a consequence of suppressed pineal and local melatonin production [1]. The emergence of amyloid-β and hyperphosphorylated tau in GBM [123, 124], as in breast cancer [125–127], may arise from the inhibition of melatonin production and the loss of capacity of melatonin to dampen inflammation and reset more optimal homeostatic interactions within a given microenvironment. As amyloid-β is an antimicrobial, its enhanced production may be a consequence of heightened brain microbial attack, including from increased circulating LPS and endogenous TLR ligands, such as HMGB1 [1]. Overall, the interactions of the P2X7r with pSTAT3 and NF-κB dimer components indicate that P2X7r significantly regulates the mitochondrial melatonergic pathway in GBM microenvironment cells, with downstream consequences on a wide array of factors linked to GBM pathophysiology, including raised levels of amyloid-β and hyperphosphorylated tau.

**Figure 6:**
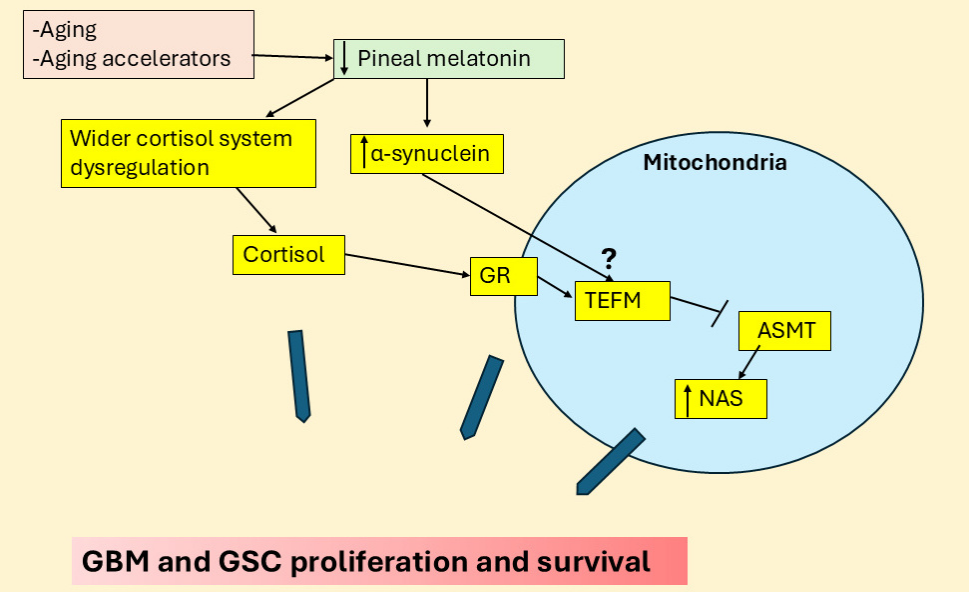
**Suppressed pineal and local melatonin increases P2X7r activation to enhance GBM survival and proliferation.** ATP: adenosine triphosphate; GBM: glioblastoma; NAS: *N*-acetylserotonin; NF-κB: nuclear factor kappa-light-chain-enhancer of activated B cells; NLRP3: NLR family pyrin domain containing 3; P2X7r: purinergic P2X7 receptor; ROS: reactive oxygen species; STAT3: signal transducer and activator of transcription 3; TLR4: toll-like receptor 4.

The high levels of α-synuclein production by GBM may also lead to P2X7r activation due to α-synuclein altering exosomal fluxes from microglia that activate the P2X7r [128]. α-Synuclein significantly suppresses extracellular ecto-ATPase activity and therefore suppresses ATP degradation, to heighten extracellular ATP and therefore P2X7r activation [129]. GBM efflux of α-synuclein also transforms the phenotypes of neighboring quiescent astrocytes to one of a stem-cell like phenotype [52]. As noted above, α-synuclein inhibits melatonin production by enhancing ASMT degradation, at least partly mediated by raised levels of autophagy associated LC3B [55]. Melatonin and α-synuclein seem to have negative reciprocal interactions as evidenced by the capacity of melatonin to suppress α-synuclein production, toxic levels and oligomerization [130–132]. The high levels of α-synuclein in GBM pathophysiology may indicate that the suppression of local melatonin production may be important to GBM survival, a process that contributes to α-synuclein induced neuronal loss in Parkinson’s disease [133]. α-Synuclein significantly interacts with the IL-6/JAK/pSTAT3 pathway [134] and NF-κB [135], indicating another route whereby α-synuclein can regulate the mitochondrial melatonergic pathway (see Figure 6).

The effects of HMGB1 in GBM include the upregulation of glycolysis under hypoxic conditions [121] and its interaction with the RAGE [136]. Glycolysis upregulation in GBM, as in other cell types, increases methylglyoxal, the effects of which are covered next.

## Methylglyoxal regulation of RAGE, pSTAT3, NF-κB, and mitochondrial function

As with most tumor cell types, increased aerobic glycolysis (Warburg effect) in GBM raises methylglyoxal levels [137]. At very high concentrations, methylglyoxal drives GBM apoptosis [138], whilst at physiological levels, methylglyoxal increases proliferation and GBM survival [72]. Much of the work on methylglyoxal has focused on its role as a precursor for the production of AGEs, which activate the RAGE [139]. Recent work indicates that methylglyoxal also depletes tryptophan, which the authors propose to be mediated by protein-protein interactions [32], indicating a suppression of tryptophan availability for the tryptophan-5-HT-NAS-melatonin pathway. Methylglyoxal may therefore further contribute to the IDO and tryptophan 2,3-dioxygenase (TDO) attenuation of tryptophan availability in GBM and the GBM microenvironment. IDO and TDO contribute to CD8^+^ T cell exhaustion [140] by converting tryptophan to kynurenine, thereby activating the AhR [141]. As indicated above, suppression of melatonin production and especially efflux in the GBM microenvironment may be an important aspect of GBM survival, given the proapoptotic effects of melatonin on GBM [9], including when used adjunctively with 50% temozolomide dose [142]. Methylglyoxal, via AGEs upregulation and protein-protein interactions with tryptophan, may contribute to melatonergic pathway suppression by a number of mechanisms.

Methylglyoxal upregulates the NF-κB p65/p50 pro-inflammatory dimer [143], which not only enhances GBM survival and proliferation and the transition of GSC [144, 145] but may also act to suppress the melatonergic pathway via interactions with nuclear pSTAT3^Tyr705^ [27]. In contrast to NF-κB p65/p50, the NF-κB c-Rel/p50 dimer composition, as shown in astrocytes, is typically anti-inflammatory and resolution inducing [146], which is proposed to be mediated at least partly via melatonergic pathway upregulation [147]. As noted above, by acting as a precursor for AGEs and RAGE activation, methylglyoxal upregulates pSTAT3 [33], which seems preferentially phosphorylated at serine727 in GBM to drive the non-canonical, mitochondria translocating pSTAT3^Ser727^ [148]. Non-canonical pSTAT3^Ser727^ can have diverse effects on mitochondrial function, including by acting at the mitochondria-associated membranes (MAMs) at the interface of mitochondria and the endoplasmic reticulum, as well as acting within the mitochondrial matrix (see Figure 3). Whether pSTAT3^Ser727^ mitochondrial translocation increases (matrix translocation) or suppresses (MAMs translocation) 14-3-3 availability for the stabilization of AANAT and the initiation of the mitochondrial melatonergic pathway requires further investigation [149]. The site of pSTAT3^Ser727^ translocation may therefore allow methylglyoxal to have differential effects on mitochondrial melatonergic pathway regulation. The diverse effects of methylglyoxal indicate that glycolysis is not simply a different/extra source of energy but is a dramatic modulator of core cellular processes pertinent to GBM initiation and proliferation.

Mitochondrial matrix translocation of pSTAT3^Ser727^ has wider consequences, including forming a positive feedback loop with the Ca^2+^ and K^+^ regulatory pore, LETMD1 [150], which is important to the pathophysiology of many types of cancer [151] and macrophage responses [152], although still to be investigated in GBM. LETM1 and its parent protein, LETMD1, have a mitochondrial matrix tail with a 14-3-3 like motif [153]. Whether AANAT or 14-3-3 and/or pSTAT3^Ser727^ can bind to this mitochondrial LETM1/LETMD1 matrix tail will be important to clarify. This could indicate that alterations in ionic regulation may be intimately coordinated with pSTAT3^Ser727^ and the positive feedback loop it forms with LETMD1, concurrent to its regulation of the mitochondrial melatonergic pathway. Overall, methylglyoxal induced pSTAT3^Ser727^ is intimately associated with alterations in mitochondrial function, patterned gene expression, and ionic regulation, as well as mitochondrial melatonergic pathway regulation.

As noted, pSTAT3^Ser727^ by translocating to MAMs modulates the crucial Ca^2+^ efflux between the mitochondrial membrane and endoplasmic reticulum membrane [154]. Mitochondrial matrix pSTAT3^Ser727^ translocation also induces the translocation of the NLRP3 inflammasome to the mitochondrial membrane, as shown in other cell types (see Figure 3), with NLRP3 activation more strongly initiated by caspases released by suboptimally functioning/challenged mitochondria, leading to raised levels of IL-1β and IL-18. In other cell types, mitochondrial pSTAT3^Ser727^ translocation also enhances NF-κB p65 mitochondrial translocation, with NF-κB and p65 modulating mitochondrial transcription and function [154] (see Figure 3). The relevance of differential effects of pSTAT3^Ser727^ to methylglyoxal and wider pSTAT3^Ser727^ inducing kinases will be important to clarify in GBM and GBM microenvironment cells.

The circadian changes over aging that interact with these processes are overviewed next.

## Integrating GBM data via pSTAT3 interaction with NF-κB dimer components

Are the putative core cellular processes in GBM highlighted above regulated by alterations in how cells and body systems are dampened and reset at night for the coming day? There is a growing appreciation of the role of circadian processes in tumor pathoetiology, including GBM [155]. Data indicates that alpha 7 nicotinic acetylcholine receptor (α7nAChR) activation suppresses GBM proliferation and survival [156], with the α7nAChR being circadian upregulated at night by melatonin, suggesting that the suppression of pineal melatonin over aging will attenuate CNS α7nAChR induction [157]. Activation of the α7nAChR inhibits the transcription of NF-κB RelA/p65 in other cell types [158] and may shift cells from a pro-inflammatory to anti-inflammatory phenotype, possibly via increasing c-Rel, either directly and/or via the induction of specialized pro-resolving mediators (SPMs) [146]. The anti-inflammatory effects of the α7nAChR may therefore be an aspect of night-time dampening and resetting across body cells/systems, at least partly mediated via an increase in SPMs and c-Rel leading to the induction of the melatonergic pathway. This is likely to have relevance not only within GBM but also in other cells of the tumor microenvironment as well as to their dynamic, homeostatic interactions. The local melatonergic pathway in GBM and GBM microenvironment cells may not be available under conditions of tryptophan depletion as induced by methylglyoxal, IDO, and TDO and/or may be altered by kynurenine activation of the AhR to increase the NAS/melatonin ratio. Overall, the suppression of pineal melatonin and its induction of the α7nAChR may have consequences for the capacity to systemically induce the mitochondrial melatonergic pathway in the course of inflammation resolution.

The suppression of pineal melatonin over aging and local melatonergic pathway suppression/alteration in GBM microenvironment cells can therefore have diverse significant consequences. Melatonin acts by a number of routes to suppress the heightened phosphorylation and activation of STAT3, with melatonin loss over aging and aging accelerating conditions, such as T2DM and obesity, disinhibiting the non-canonical pSTAT3^Ser727^ effects in GBM mitochondria, with potential consequences for all GBM microenvironment cells. As pSTAT3^Ser727^ alters MAMs/Ca^2+^ and LETM1/LETMD1/Ca^2+^ and K^+^, its mitochondrial translocation can have significant impacts on ionic regulation in mitochondria. Mitochondrial ionic regulation is a core aspect of mitochondrial function, including in the modulation of ROS and ROS-dependent miRs and therefore patterned gene expression among cells in GBM and therefore the interactions of cells in the GBM microenvironment. The aging-linked decreased availability of pineal melatonin, by disinhibiting pSTAT3^Ser727^ will also increase the mitochondrial translocation of the NLRP3 inflammasome and therefore levels of the pro-inflammatory cytokines, IL-1β and IL-18, as well as the mitochondrial translocation of NF-κB and its pro-inflammatory component, p65 [154]. Such data in other cell types indicates how an inflammatory milieu may become established via alterations in night-time associated processes. The investigation of the changing nature of the melatonergic pathway in all GBM microenvironment cells should better clarify the relevant dynamic homeostatic alterations occurring in the pathoetiology and ongoing pathophysiology of GBM and its interactions with other GBM microenvironment cells.

Astrocytes have an enhanced p65/p50 NF-κB dimer expression during inflammation, which is gradually replaced by c-Rel/p50 in the shift to an anti-inflammatory phenotype [146]. These differential effects of NF-κB dimer composition are evident across a number of different cell types [159–162]. However, there is some variability across cell types as to how NF-κB dimer composition can modulate the melatonergic pathway [27], indicating the need for future research to clarify the role of the NF-κB dimer composition in specific cells. Consequently, there is a growing appreciation of a more nuanced role of NF-κB dimer composition in immune cell regulation [163]. Whether NF-κB p65/p50 suppresses the melatonergic pathway in GBM, astrocytes, microglia, and other cells of the tumor microenvironment will be important to determine, including how this changes over the course of aging. Data shows these aging-associated changes in NF-κB to occur in microglia [164], and therefore may be pertinent to how aging may increase GBM susceptibility via the modulation of other cells in the tumor microenvironment. The dynamic interactions of GBM with astrocytes and microglia are clearly important in the modulation of treatment response [165], and their differential circadian regulation over aging will be important to clarify.

There is a growing appreciation of the potential role of SPMs in tumor pathophysiology and management, given their powerful role in the regulation of inflammatory processes, including in GBM [166]. Enhanced inflammation is a risk factor for most cancers, as is angiogenesis, both of which are inhibited by SPMs, allowing SPMs to decrease tumor growth and improve treatment [167–169]. How SPMs are integrated into the homeostatic interactions of the GBM tumor microenvironment will be important to determine, including the role of ACh or kynurenic acid activation of the α7nAChR in upregulating SPMs. The SPM, neuroprotectin D1, upregulates c-Rel and therefore may disinhibit local melatonin production in the course of night-time dampening and resetting. This requires future investigation in the GBM microenvironment.

**Figure 7:**
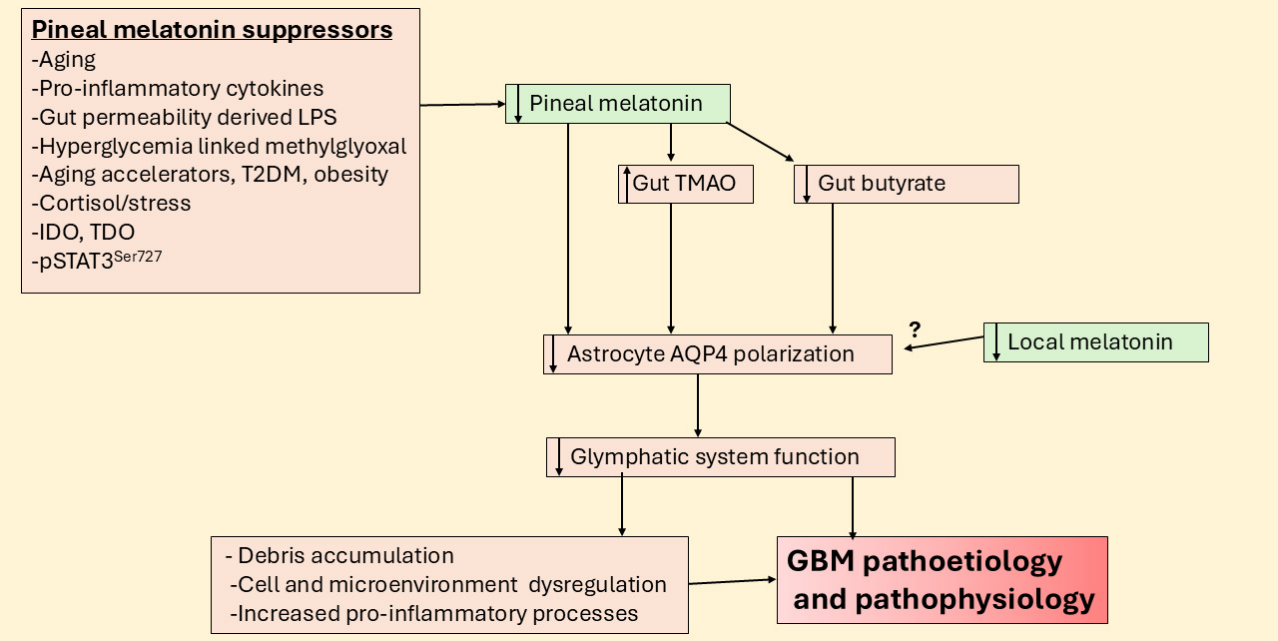
**Pineal suppressors act on CNS and systemic processes to initiate GBM.** GBM: glioblastoma; IDO: indoleamine 2,3-dioxygenase; LPS: lipopolysaccharide; NF-κB: nuclear factor kappa-light-chain-enhancer of activated B cells; pSTAT3: phosphorylated signal transducer and activator of transcription 3; T2DM: type 2 diabetes mellitus; TDO: tryptophan 2,3-dioxygenase.

This is given some support by data showing GBM patients to have a significant decrease in circulating kynurenine, 5-hydroxytryptophan, and 5-HT, coupled to a trend decrease in circulating tryptophan [170], with the suppression of these factors correlated with decreased survival time. Whether this indicates the suppression of the tryptophan-5-HT-NAS-melatonin pathway, including by methylglyoxal, in the course of the Warburg effect and/or by decreased pineal melatonin over aging and the dysregulation of the wider cortisol system and/or increased pSTAT3^Ser727^ and/or from increased gut permeability and gut dysbiosis, including decreased epigenetic regulation by butyrate, will be important to clarify. Alterations in the melatonergic pathway are intimately linked with many of the putative core processes classically investigated in GBM, including the role of a disinhibited wider cortisol system in the course of night-time dampening and resetting, as well as the role of gut dysbiosis/permeability. Clearly, the classical role of increased AhR activation across cancer types is intimately linked to alterations in the regulation of the melatonergic pathway via AhR/CYP1B1/CYP1A2 driving the *O*-demethylation of melatonin to NAS [26, 171], potentially providing trophic support to GBM via TrkB activation [24]. For example, whether the GBM efflux of kynurenine that activates the AhR on NK cells and CD8^+^ T cells not only drives the ‘exhaustion’ of these cytolytic cells but also increases NAS release to enhance the proliferation and survival of GSC [56] will be important to clarify. Variations in melatonin availability over aging and aging-accelerating conditions modulate a number of core processes intimately associated with STAT3 and NF-κB regulation and interaction, which are highlighted in Figure 7.

This has a number of future research and treatment implications.

## Future research directions

Does melatonin induction of miR-138 suppress PD-1 expression in the GBM microenvironment?

Does cortisol at the mitochondrial GR induce TEFM to increase the proliferation and survival of GBM [44] via ASMT suppression? Does this have any relevance to the effects of α-synuclein, which can also suppress ASMT availability?

Does P2X7r activation in GBM and GBM microenvironment cells increase NAS and the NAS/melatonin ratio, as evident in pinealocytes [113]? This may be important given the capacity of NAS to activate TrkB [24], with TrkB activation increasing the survival and proliferation of GSC [58].

Does GBM α-synuclein production lead to P2X7r activation by altering exosomal fluxes from microglia that activate the P2X7r [128]?

Does non-canonical pSTAT3^Ser727^ limit mitochondrial 14-3-3 availability to stabilize AANAT? Is this regulated by pSTAT3^Ser727^, having a positive feedback loop with LETMD1?

Do AANAT or 14-3-3 and/or pSTAT3^Ser727^ bind to the mitochondrial LETM1/LETMD1 matrix tail, thereby coordinating pSTAT3^Ser727^ and AANAT/melatonin regulation with Ca^2+^ and K^+^ regulation in GBM? Given the close proximity of this matrix tail with mitochondrial ribosomes, does this have implications for mitochondrial patterned gene induction? Is LETMD1 expressed on the GBM mitochondria membrane in correlation with pSTAT3^Ser727^ levels?

Are the GR-α and/or GR-β expressed on the mitochondrial membrane and/or mitochondrial matrix in GBM, thereby altering the regulation of mitochondrial function that seems important to GBM pathophysiology? Is any alteration in GR expression at mitochondria determined by circadian alterations over aging, including the suppression of pineal melatonin?

## Prevention

As aging is the major risk factor for GBM, understanding the circadian, systemic, and cellular processes driving aging-linked physiological changes is clearly an important goal. Given the putative importance of the loss of pineal melatonin over aging in the pathoetiology of aging-linked conditions, such as GBM, the targeted increase in pineal melatonin production will be an important preventative target for GBM, as for other aging-associated medical conditions.

Factors that suppress aging-associated changes, such as EGCG, which inhibits the AhR as well as maintains the gut barrier and suppresses monoamine oxidase to increase 5-HT availability as a precursor for the melatonergic pathway, will afford some protection against GBM [1].

Hyperglycemia, and associated increased methylglyoxal, is a GBM risk factor and increases GBM survival and proliferation, whilst decreasing apoptosis [172, 173]. Diet and exercise modulate hyperglycemia, prediabetes, and T2DM to decrease GBM risk.

Quercetin quenches and directly binds methylglyoxal [174] as well as having wider utility in the treatment of established GBM [175], including the suppression of GSC [176]. Increased methylglyoxal is likely relevant to the putative causal relationship between T2DM and GBM [71] and therefore likely to derive benefit from quercetin. The benefits of quercetin include the inhibition of the IL-6/JAK/pSTAT3 pathway in GBM, which increases GBM apoptosis and decreases metastasis [177], suggesting that the efficacy of quercetin may involve the regulation of the melatonergic pathway.

## Treatment

The holy grail of most cancer research is the capacity to turn tumorous proliferating cells into differentiated cells. This has been achieved to some degree in GBM cells by the combination of Y27632, forskolin, SB431542, and SP600125, which are proposed to selectively target ROCK, cAMP, TGF-β, and JNK signaling pathways, respectively [178]. Whether this can be refined by incorporating night-time processes and circadian regulation will be important to determine in vivo. For example, forskolin is long appreciated to increase AANAT and the melatonergic pathway [179], whilst melatonin decreases TGF-β [180] and has regulatory effects on JNK and ROCK [181, 182] as well as increasing the apoptosis and differentiation of numerous tumor cell types [183, 184], including GBM [56, 185]. This would indicate that incorporating the role of the melatonergic pathway as an aspect of core cell functioning will inform us as to the relevant processes underpinning treatment strategies.

As with many tumor types, there has been extensive investigation of the role of immune checkpoint inhibitors in the treatment of GBM, including T cell immunoglobulin and mucin domain 3 [186], cytotoxic T-lymphocyte associated protein 4 [187] and PD-1 [188], invariably with limited clinical effects even when used in combination [189]. As well as killing cancer cells, melatonin also increases the efficacy of tumor-suppressing immune cells, including increasing the cytotoxicity of NK cells [41, 43], indicating its utility as an adjunctive to immune checkpoint inhibitors [190].

Chimeric antigen receptor-T cell therapies are a more promising treatment that also target the immune response, with some evidence to indicate the clinical utility of adjunctive melatonin, especially in the prevention of cytokine release syndrome, which is a common side-effect of chimeric antigen receptor-T cell therapies [191]. Chimeric antigen receptor-T cell therapies have been evaluated in phase I clinical trials with GBM patients and clearly need to be refined to improve clinical efficacy [192]. Whether chimeric antigen receptor-T cell and immune checkpoint inhibitor therapies have any modulator effects on the tryptophan-5-HT-NAS-melatonin pathway will be important to clarify in tumor cells and other cells of the tumor microenvironment and may contribute to the refinement of treatment strategies.

The gut microbiome-derived butyrate is a pan-HDACi, with HDACi currently at the forefront of cancer treatment development [193]. Other HDACi, such as sodium valproate, increase NKG2D in GBM to increase the efficacy of NKG2D targeting chimeric antigen receptor-T cell therapy [194]. Butyrate also suppresses GR-α nuclear translocation and therefore will suppress some of the protumor effects arising from wider cortisol system dysregulation over the course of aging.

A ketogenic diet mediates its effects via butyrate upregulation in preclinical glioma models, which the authors attribute to the induction of caspase 3 in microglia, leading to an anti-glioma microglia phenotype [195]. These authors also showed butyrate and butyrate producing microbiota were decreased in glioma patients, highlighting the role of the gut microbiome in the modulation of the GBM tumor microenvironment [195]. GBM associated microglia can show both M1-like and M2-like phenotypes, indicating their complex role and plasticity in the GBM microenvironment [196]. As the induction of the melatonergic pathway via STAT3 interactions with NF-κB dimer composition determines the shift from an M1-like to M2-like phenotype [197], the regulation of the microglia melatonergic pathway should clarify the microglia’s role in GBM and provide future treatment targets.

Melatonin pretreated mesenchymal stromal cells significantly decrease GBM proliferation and metastasis, as shown in a preclinical GBM xenograft murine model [198], highlighting the relevance of variations in melatonin in the modulation of responses amongst cells of the tumor microenvironment.

The polyphenol, EGCG, decreases GBM survival and metastasis [199], as well as inhibiting the protumor effects of local cortisol production by inhibiting 11β-HSD1 via protein-protein interactions [46]. EGCG, like curcumin, resveratrol, folate, and vitamin B12, also inhibits the AhR [200], and therefore can increase the cytotoxicity of NK cells and CD8^+^ T cells, as well as suppress AhR activation in the regulation of the melatonergic pathway.

The ring finger protein, RNF213, is a tumor suppressor. Higher RNF213 levels are associated with increased GBM patient survival [201]. Decreased or mutated RNF213 increases both the tumor proliferation and the microvascular expansion [202], which is mediated by an increase in the JAK/STAT3 pathway [203], potentially implicating alterations in the mitochondrial melatonergic pathway. RNF213 may be regulated by a number of nutriceuticals, including EGCG and resveratrol, with differential binding affinities of these nutriceuticals in mutated RNF213 [204]. Targeting RNF213 upregulation across GBM microenvironment cells, including by nutriceuticals, should increase GBM patient survival and requires investigation into whether this is driven by regulation of the STAT3 and NF-κB dimer interactions in modulating the melatonergic pathway in GBM microenvironment cells.

**Figure 8:**
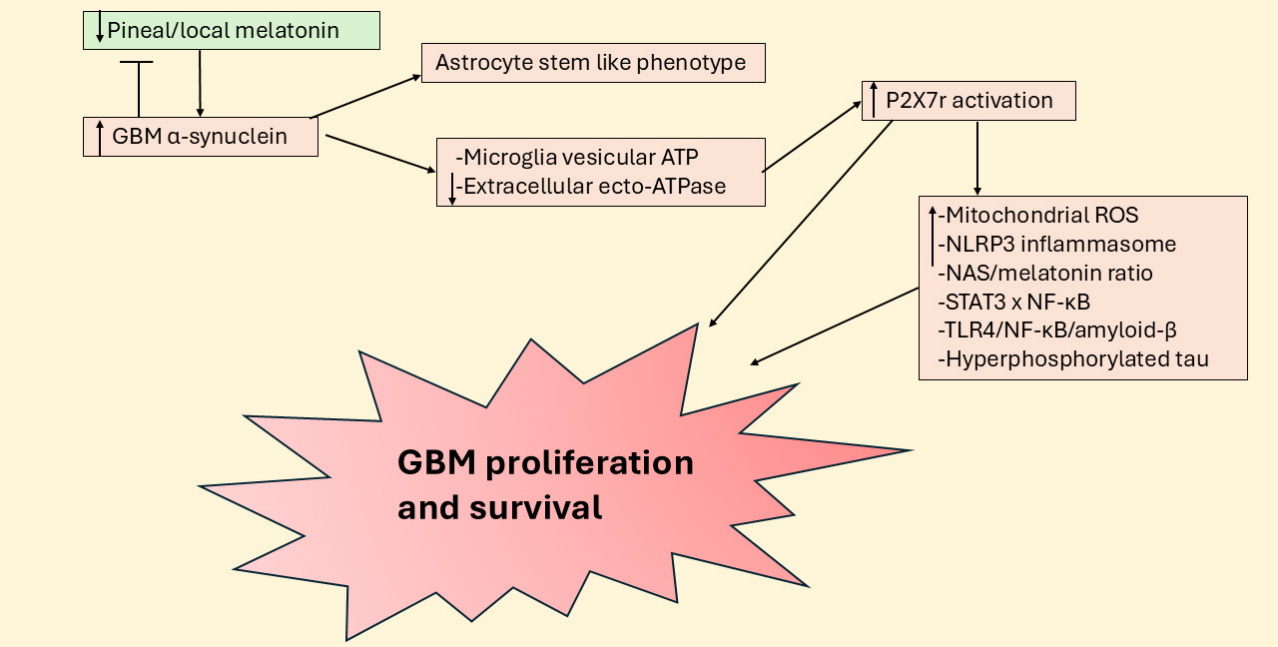
**TMS may act via pineal melatonin, oxytocin, and vagal nerve in GBM.** ACh: acetylcholine; GBM: glioblastoma; IDO: indoleamine 2,3-dioxygenase; IL: interleukin; NF-κB: nuclear factor kappa-light-chain-enhancer of activated B cells; PVN: paraventricular nucleus; SPMs: specialized pro-resolving mediators; STAT3: signal transducer and activator of transcription 3; TDO: tryptophan 2,3-dioxygenase; TMS: transcranial magnetic stimulation; α7nAChR: alpha 7 nicotinic acetylcholine receptor.

Vagal nerve stimulation shows some promise in GBM management, which is proposed to be mediated by a decrease in systemic pro-inflammatory processes and factors, including raised levels of systemic IL-6 [205]. IL-6 is a major driver of the JAK/STAT3 pathway and intimately associated with NF-κB activation in GBM, with STAT3 transcriptionally upregulating PD-L1 [206]. As pro-inflammatory NF-κB p65 is increased in GBM and drives proliferation [207], the upregulation of PD-L1 is likely to be associated with melatonergic pathway suppression. Vagal suppression and the correlated IL-6 upregulation may therefore be intimately linked to proliferative processes and the suppression of the GBM melatonergic pathway. Melatonin increases oxytocin to activate the vagal nerve and dampen systemic inflammatory processes, with effects at least partly mediated by vagal ACh/α7nAChR/SPMs/NF-κB dimer component changes that increase local melatonin [208]. The suppressed levels of pineal melatonin attenuate its capacity to upregulate oxytocin and therefore the oxytocin stimulation of the vagal nerve. This leads to an attenuated capacity of the vagal nerve to dampen systemic inflammatory processes. As the efficacy of vagal stimulation to dampen inflammatory processes, as shown in the gut, is dependent upon the upregulation of the local melatonergic pathway [208], local melatonin regulation in a given organ/tissue determines vagal stimulation efficacy. Such data on oxytocin is in contrast to its effects in GBM cell lines, where it is proposed to have proliferative effects [209] and highlights the importance of understanding holistic, systemic processes and not simply the end-point chaos of isolated GBM cell lines. This is further highlighted by the impacts of transcranial magnetic stimulation (TMS) in GBM management (see Figure 8).

Data indicate some utility of TMS in the management of GBM [210]. As TMS over the frontal lobes increases pineal melatonin [211] and suppresses cortisol and GR-α activation [212], TMS efficacy in GBM (and many other medical conditions) may be mediated via the suppression of the aging-associated decrease in the night-time melatonin/cortisol ratio, with consequent impacts on how systemic and CNS processes are dampened and reset at night. Given the integration of GBM with the circadian rhythm of CNS cells, alterations in circadian processes are likely to indicate changes in GBM interactions with other microenvironments and wider CNS cells. TMS also increases glymphatic system function, as shown in preclinical models [213], including in brain parenchyma and the meningeal lymphatics, indicating wider benefits on aging-associated deficits in glymphatic system function, including in GBM pathoetiology [214]. As to whether this is mediated by the beneficial effects of pineal (and/or local) melatonin production and/or suppression of cortisol and GR-α activation by TMS requires further investigation.

Chimeric antigen receptor NK cell therapy is developing treatment for many tumors, including GBM, where it is proposed that shaping the GBM microenvironment to a less immune suppressed state may enhance chimeric antigen receptor NK cell therapy [215]. Recent work indicates that NK cells may express a mitochondrial melatonergic pathway, allowing for a targeted upregulation of the NK cell melatonergic pathway, whereby released melatonin has autocrine effects that enhance NK cell cytotoxicity, whilst also having paracrine effects on GBM to decrease their survival and metastasis [216]. The investigation of the melatonergic pathway may therefore have significant effects on near-future treatment developments.

## Conclusions

GBM is a complex condition that is poorly understood and poorly treated, with a median life expectancy at diagnosis of less than two years. Consequently, a conceptualization of GBM pathoetiology and pathophysiology that integrates wider bodies of data collected in this condition is clearly required. Incorporating the interactions of two factors strongly associated with GBM pathophysiology, namely canonical and noncanonical STAT3 and NF-κB dimer composition, and their interactions in the regulation of the mitochondrial melatonergic pathway provides a novel conceptualization of GBM pathoetiology and pathophysiology, with numerous future research, prevention, and treatment implications. GBM is an aging-associated condition with an etiology that seems powerfully determined by the dramatic decrease in pineal melatonin over aging and in aging-accelerating conditions, which changes the effects of circadian and stress cortisol, thereby altering how systemic cells, microenvironments, and body systems are prepared for the coming day. As with other ‘inflammaging’ linked conditions, the capacity to achieve resolution and a return to homeostatic intercellular interactions in a given microenvironment is attenuated by the decrease in the melatonin/cortisol night-time ratio. The attainment of homeostatic intercellular resolution is strongly determined by the capacity to shift STAT3 and NF-κB interactions to one that increases local melatonin release. As indicated throughout, many previously disparate bodies of GBM data may be integrated within this basic perspective, which clearly requires future investigation.

## Abbreviations

11KT: 11-ketotestosterone
5-HT: serotonin
AANAT: aralkylamine *N*-acetyltransferase
ACh: acetylcholine
AGEs: advanced glycation end products
AhR: aryl hydrocarbon receptor
AQP4: aquaporin 4
ASMT: *N*-acetylserotonin *O*-methyltransferase
BDNF: brain-derived neurotrophic factor
CAR: cortisol awakening response
COX: cyclooxygenase
CYP: cytochrome P450
EGCG: epigallocatechin gallate
GBM: glioblastoma
GR: glucocorticoid receptor
GSC: glioblastoma stem-like cell
HDACi: histone deacetylase inhibitor
HMGB1: high mobility group box 1
HPA: hypothalamic-pituitary-adrenal
IDO: indoleamine 2,3-dioxygenase
IL: interleukin
JAK: Janus kinase
LC3B: light chain 3 beta
LETMD1: leucine zipper EF-hand containing transmembrane protein-domain containing 1
LPS: lipopolysaccharide
LRRK2: leucine-rich repeat kinase 2
MAMs: mitochondria-associated membranes
miR: microRNA
NAD^+^: nicotinamide adenine dinucleotide
NAS: *N*-acetylserotonin
NF-κB: nuclear factor kappa-light-chain-enhancer of activated B cells
NK: natural killer
NLRP3: NLR family pyrin domain containing 3
P2X7r: purinergic P2X7 receptor
PARP1: poly-ADP-ribose polymerase 1
PD: programmed cell death
PD-L1: programmed cell death-ligand 1
PG: prostaglandin
pSTAT3: phosphorylated signal transducer and activator of transcription 3
RAGE: receptor for advanced glycation end products
ROS: reactive oxygen species
SPMs: specialized pro-resolving mediators
STAT3: signal transducer and activator of transcription 3
T2DM: type 2 diabetes mellitus
TDO: tryptophan 2,3-dioxygenase
TEFM: mitochondrial transcription elongation factor
TLR4: toll-like receptor 4
TMS: transcranial magnetic stimulation
TrkB: tyrosine receptor kinase B
α7nAChR: alpha 7 nicotinic acetylcholine receptor

## Declarations

### Copyright

© The Author(s) 2026.
